# The Utilization of Freezing Steps in Mesenchymal Stromal Cell (MSC) Manufacturing: Potential Impact on Quality and Cell Functionality Attributes

**DOI:** 10.3389/fimmu.2019.01627

**Published:** 2019-07-16

**Authors:** Sofia Oja, Tanja Kaartinen, Marja Ahti, Matti Korhonen, Anita Laitinen, Johanna Nystedt

**Affiliations:** Advanced Cell Therapy Centre, Finnish Red Cross Blood Service, Helsinki, Finland

**Keywords:** cryopreservation, freezing, MSC, mesenchymal stromal cell, immunosuppression, cell banking, master cell bank

## Abstract

Some recent reports suggest that cryopreserved and thawed mesenchymal stromal cells (MSCs) may have impaired functional properties as compared to freshly harvested MSCs from continuous cultures. A cryopreservation step in the manufacturing process brings important benefits, since it enables immediate off-the-shelf access to the products and a completion of all quality testing before batch release and administration to the patient. Cryopreservation is also inevitable in MSC banking strategies. In this study, we present the results from the MSC stability testing program of our in-house manufactured clinical-grade allogeneic bone marrow-derived MSC product that is expanded in platelet lysate and frozen in passage 2. The current manufacturing protocol contains only one freezing step and the frozen MSC product is thawed bed-side at the clinic. We can conclude superior viability and cell recovery of the frozen and thawed MSC product utilizing the validated freezing and thawing protocols we have developed. The MSC phenotype and differentiation potential was generally found to be unaltered after thawing, but the thawed cells exhibited a 50% reduced, but not completely abolished, performance in an *in vitro* immunosuppression assay. The *in vitro* immunosuppression assay results should, however, be interpreted with caution, since the chosen assay mainly measures one specific immunosuppressive mechanism of MSCs to suppress T-cell proliferation. Since at least two freezing steps are usually necessary in MSC banking strategies, we went on to investigate the impact of repeated freezing on MSC quality attributes. We can conclude that two freezing steps with a preceding cell culture phase of at least one passage before freezing is feasible and does not substantially affect basic cell manufacturing parameters or quality attributes of the final frozen and thawed product. Our results suggest, however, that an exhaustive number of freezing steps (≥4) may induce earlier senescence. In conclusion, our results support the utilization of frozen MSC products and MSC banking strategies, but emphasize the need of always performing detailed studies on also the cryopreserved MSC counterpart and to carefully report the cryopreservation and thawing protocols.

## Highlights

- 1-2 freezing steps for MSCs in early passage is feasible and preserves most of the *in vitro* functional properties.Interim freezing steps are not reflected in standard manufacturing parameters.
- the *in vitro* immunosuppressive performance of frozen and thawed MSCs may be different from their fresh counterparts with a reduced, but not abolished *in vitro* performance specific for the IDO pathway.
- *in vitro* immunosuppression assay results ought to be interpreted with caution.
- cryopreserved and thawed MSCs may be different from their fresh counterparts, but that does not necessarily translate to reduced clinical efficacy.

## Introduction

Mesenchymal stromal cells (MSCs) are being widely studied as potential cell therapy medicinal products due to their immunomodulatory properties, which have been established by *in vitro* studies and in several clinical trials (1, 2). Within this context, MSC therapy may hold substantial promise particularly in the treatment of inflammatory and autoimmune conditions and MSCs have therefore been widely employed as a salvage treatment option in refractory graft-vs.-host disease (GvHD) in its acute form (3–6). It is, however, becoming evident that albeit some patients with severe acute GvHD markedly benefit from the MSC treatment, some patients experience no improvement of the symptoms (7, 8). Based on numerous published patient cohorts and thousands of treated patients, the safety of MSC therapy appears clear, but less certain is the efficacy of the MSC therapy. It is currently evident that the overwhelming positive results reported from *in vitro* and preclinical animal studies have largely not yet translated into full clinical efficacy.

Clearly, there is still much to be learned and optimized with regards to the *in vivo* interactions of MSCs in human pathological states. It has been thought that allogeneic MSCs do not provoke an overt immune reaction in the host even when the host and donor are not human leukocyte antigen (HLA) matched. This concept has been challenged recently, but fortunately not from a safety point of view. Allogeneic MSCs are obviously not as hypoimmunogenic as originally thought and an immune activation of host cytotoxic T-cells and cytotoxic activity against MSCs is actually critical for effective immunosuppression through phagocytosis of apoptotic MSCs and subsequent macrophage polarization (9, 10). The apoptosis-based MSC immunomodulation mechanism has significantly improved our understanding on the mechanistic properties of MSCs, but we also need to clarify how the functional properties of MSCs may be affected by differences in the manufacturing strategies and culturing conditions.

Clinical MSC preparations can either be fresh, meaning the cells are detached from the cell cultures just before administration to patients, or the cells can be cryopreserved and thawed bedside just before administration. Naturally, every step of the clinical manufacturing process need to fulfill local legislation, such as the Advanced Therapy Medicinal Product (ATMP) legislation in all EU countries, and comply with GMP requirements specific for the area. MSCs are usually manufactured from third-party healthy donors and administered in a completely HLA-mismatched allogeneic setting. Not unsurprisingly, it is therefore more common to utilize cryopreserved MSCs in the clinical setting, since cryopreservation includes several obvious benefits: (1) rapid access to the cell product in acute conditions, (2) convenient logistics to the clinical site, (3) minimal reconstitution activities of the cell product bed-side, (4) substantial quality benefits with all quality testing completed before batch release and administration to the patient, (5) large-scale manufacturing and master cell bank opportunities, and (6) enablement of treatment protocols with numerous identical cell doses. The effect of cryopreservation on MSC therapeutic properties has, however, become highly controversial. Some recent reports suggest that cryopreserved MSCs may have impaired functional properties when compared with freshly harvested MSCs from continuous cultures (11–13). On the contrary, some recent conflicting studies have shown that the functionality and cell characteristics of cryopreserved MSCs are comparable to fresh MSCs (14–16). Cryopreserved MSCs have been explored in several clinical studies for graft-vs.-host disease by us and others with partially encouraging results (7, 17–21). These conflicting results warrant further studies to elucidate the impact of a cryopreservation step in the manufacture of clinical-grade MSCs.

In this study, we performed an in-depth analysis of our in-house manufactured and cryopreserved platelet lysate-expanded bone marrow-derived MSC product, which is provided frozen in passage 2 (p2) for clinical use. We report superior viability and cell recovery of our cryopreserved MSC product utilizing the freezing and thawing protocols we have established. The MSC phenotype and differentiation potential was generally unaltered after thawing, but we report a reduced, but not abolished, *in vitro* immunosuppression capacity directly after thawing. Since our current manufacturing protocol contains only one freezing step in p2 and multiple freezing steps are usually necessary in MSC banking strategies, we went on to investigate the impact of repeated freezing. We can conclude that two freezing steps with preceding cell culture of at least one passage before freezing is feasible and does not substantially affect basic cell manufacturing parameters (cell yield, growth kinetics, population doubling (PD) number) or the basic quality attributes of the final frozen and thawed product. Our results indicate that freezing of MSCs is feasible and preserves most of the functional properties of the product, keeping in mind that the *in vitro* immunosuppressive potency assay results with thawed MSCs could indicate a reduced IDO-dependent immunomodulatory capacity. Our results might also suggest that an exhaustive amount of freezing steps (≥4) may accelerate the induction of senescence.

## Materials and Methods

### MSC Starting Material

Healthy, voluntary bone marrow (BM) donors were recruited specifically for donating starting material for clinical-grade MSC manufacturing. The donor recruitment, eligibility assessment and bone marrow aspiration protocol was approved by the local ethics committee and the BM collection procedure was authorized by the Finnish Medicines Agency under a tissue establishment license. Forty milliliters of bone marrow was aspirated under local anesthesia from the posterior iliac crest into heparinized syringes after a written informed consent. Donor eligibility was assessed by an extensive health questionnaire and testing for infectious diseases (NAT tests: HIV-1, HBV, and HCV by Ultrio NAT, B19 (Parvo), HAV; Serological tests: HBsAg, HCVAb, HIVAb/Ag, HBcAb, TrpaAb (Syphilis), HTLVAb and CMVAbG). The donors had an average age of 25 years (range 18–35 years) and the male/female ratio was 30/70%, respectively.

### Bone Marrow-Derived MSC (BM-MSC) Culture Protocol

**Figure 1:**
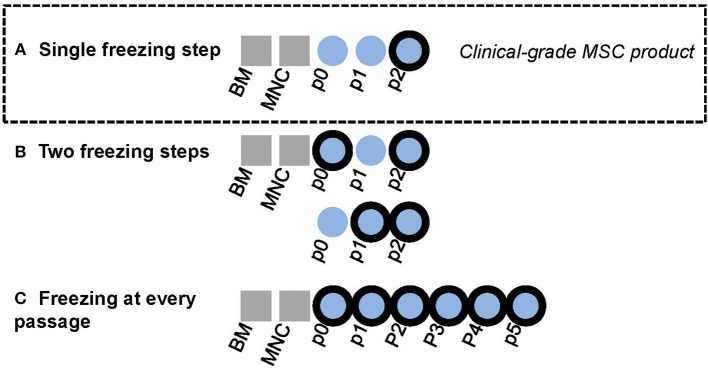
Graphical layout of experimental design. Bold black outliners indicate freezing steps. **(A)** In-house BM-MSC manufacturing protocol for clinical-grade MSC product. The final product in passage two is cryopreserved. **(B)** The effect of adding one interim freezing step at either passage 0 or passage 1 in addition the final freezing step at passage 2 was explored in this study. **(C)** Some MSC lines were cultured until passage 5 in this study with freezing at every passage.

The clinical-grade MSC culture protocol layout is presented in Figure 1 and has been previously published together with clinical data (7, 8). Cultured MSCs are classified as ATMPs in the EU and the clinical-grade MSC products were manufactured in the GMP facility of the Finnish Red Cross Blood Service in Helsinki under a national ATMP hospital exemption license authorized by the Finnish Medicines Agency. The BM sample was processed within 2 h of aspiration and was diluted with DPBS (CTS Life Technologies, Thermo Fischer Scientific, MA, USA) containing 0.02% Versene (EDTA, Lonza, Switzerland) before density gradient centrifugation (Ficoll-Paque Premium, GE Healthcare Biosciences, Sweden). Primary cultures (p0) were initiated by plating isolated BM mononuclear cells (MNCs) at 400 000 cells/cm^2^. All culturing steps were performed at +37°C and in humidified atmospheric oxygen with 5% CO2. The animal serum-free, antibiotic-free basal culture medium consisted of D-MEM low glucose (Life Technologies) supplemented with 40 IU/ml heparin (Heparin Leo 5000 IU/ml, Leo Pharma, Sweden) and 10% pooled platelet lysate (Finnish Red Cross Blood Service, Helsinki, Finland; for more details see (22, 23). Culture medium was replaced twice a week and the cells were detached with TrypLE Select CTS (Life Technologies). The cells were plated at a density of 1000 cells/cm^2^ from p1 and onwards. Culture confluency and cell morphology were monitored by phase contrast microscopy. Cell numbers and viability were determined using the NucleoCounter NC-100 (ChemoMetec A/S, Denmark). Research-grade MSC cultures (from p3 onwards) were cultured outside the GMP facility and the cultures were additionally supplemented with 100 IU/ml Penicillin and Streptomycin (Gibco, Life Technologies).

### Freezing and Thawing of MSCs

Clinical-grade MSCs in passage 2 were detached from cultures with TrypLE Select CTS (Life Technologies), washed with DPBS and suspended in a pre-chilled freezing medium consisting of 10% dimethylsulfoxide (DMSO, CryoSure, Wak-Chemie Medical GmbH, Germany) and 90% human serum albumin (Albunorm 200 g/l, Octapharma, Switzerland). Clinical-grade cells were dispensed in freezing bags with either 50 × 10^6^ or 100 × 10^6^ cells/bag (CryoMACS®, Miltenyi Biotech GmbH, Germany) as described previously (7). Research-grade cultures were frozen in smaller cryovials (Nunc CryoTubes®, Nalge Nunc, NY, USA), but with comparable cell density. Both freezing bags and cryovials were slowly chilled at −70°C before transferring the bags or vials to vapor (clinical-grade) or liquid (research-grade) nitrogen tanks at ≤ −140°C. Frozen bags or cryovials were thawed at +37°C water bath according to a standardized operating procedure. The exact thawing time was always documented and never exceeded 3 min. Clinical-grade cells in freezing bags were always diluted in saline to 2 × 10^6^ cells/ml immediately after thawing, after which the thawed cells were filtered through a 200 μm clinical filter. The cells were allowed to rest at room temperature for 5 to 15 min before analysis or further processing.

### Colony Forming Units and Population Doubling Numbers

Original MSC amounts in the bone marrow aspirate was determined by the colony forming units-fibroblast (CFU-F) assay (24). Four Lakhs isolated BM-MNCs/cm^2^ were plated in replicate on 6-well plates in basal culture medium and were let to adhere for 72 h. Non-adherent cells were removed, the wells were washed four times with DPBS and culturing was continued for 5–10 days until individual colonies consisting of at least 20 cells were clearly visible. The cells were fixed with methanol (Methanol EMSURE® ACS, Merck KgAa, Germany), stained with Giemsa stain (Giemsa's Azur Eosin Methylene Blue, Merck, KgAa) and the number of colonies in each well was calculated. The CFU-F colony numbers were correlated to the plated MNC numbers and culture area cm^2^. Population doubling (PD) numbers were calculated for each passage according to the equation [PD = log^2^ (NH/N1)], where N1 is the number of seeded cells/cm^2^, and NH is the number of harvested cells/cm2. The CFU-F number of the p0 culture was used as the first N1 (22). The cumulative PD number thus also include the amount of population doublings that occur during the initial p0 culture stage.

Cell cultures were considered fully senescent when the confluency of the culture remained under 30% after 2 weeks of culturing without passaging combined with manifested senescence-associated morphological characteristics such as enlarged cell size and granularity (25).

### Cell Surface Phenotype by Flow Cytometry

The MSC immunophenotype was determined by flow cytomety using the Navios Cytometer (Beckmann Coulter, IN, USA) and FlowJo analysis software (version 7.4.1. TreeStar Inc., CA, USA). The cell surface antigens CD44, CD49e, CD13, CD90, CD73, CD29, CD86, CD105, HLA-ABC, CD14, CD19, CD34, CD45, CD80, and HLA-DR were analyzed and at least 5000 cells were included in the analysis. All antibodies were purchased from BD Biosciences (CA, USA) except CD80 (Beckmann Coulter) and HLA-DR (Abcam, UK). The proportion of non-viable cells were determined during analysis by staining the cells with propidium iodide (50 μg/ml in PBS, BD Biosciences).

### Differentiation Assays

#### Osteogenic Differentiation Assay

MSCs were plated at a density of 1000 cells/cm^2^ in 6-well-plates and cultured in platelet lysate supplemented basal culture medium with 100 IU/ml penicillin and streptomycin until 70% confluency. Osteogenic differentiation was induced with 0.1 μM dexamethasone (Dexamethasone, BioXtra, Sigma, MO, USA), 50 μM ascorbic acid (Ascorbic acid 2-phosphate, Sigma) and 10 mM β-glyserophosphate (β-Glyserophosphate disodium salt pentahydrate, AppliChem, Germany). The MSCs were maintained in the induction medium until the formation of visible calcium phosphate precipitate. The cells were fixed with 4% paraformaldehyde (PFA) and the calcium phosphate precipitate was stained using von Kossa staining.

#### Adipogenic Differentiation Assay

MSCs were plated at density 1000 cells/cm^2^ in 6-well plates and cultured in the platelet lysate supplemented basal culture medium with 100 IU/ml penicillin and streptomycin until 70–100% confluent. Adipogenic differentiation was induced for 3–4 days by an induction medium consisting of αMEM GlutaMax (Gibco, Life Technologies), 10% inactivated fetal bovine serum (Gibco, Life Technologies), 20 mM Hepes (Gibco, Life Technologies), 100 U/ml penicillin and streptomycin (Gibco, Life Technologies) supplemented with the induction cocktail of 0.1 mM indomethacin (Sigma), 44 μg/ml 3-isobutyl-methyl-xanthine (IBMX-22^*^), 0.5 μg/ml insulin (Insulin-0.25^*^) and 0.4 μg/ml dexamethasone (DM-200^*^) (^*^Preadipocyte Differentiation Medium Supplement Pack, PromoCell, Italy). Control cells were only maintained in induction medium without the induction cocktail. Differentiation was finalized by culturing the cells in a terminal differentiation medium consisting of induction medium supplemented with 0.1 mM indomethacin (Sigma), 0.5 μg/ml insulin (Insulin-0.25^*^) and 3.0 μg/ml ciglitazone (Ciglitazone-1.5^*^) (^*^Preadipocyte Differentiation Medium Supplement Pack) for 2–4 weeks until visible lipid droplets could be observed. The cells were fixed with 4% PFA and were stained using Sudan III.

### Immunosuppression Assays

#### Basic T-Cell Proliferation Assay

Peripheral blood mononuclear cells (PBMNCs) from healthy voluntary blood donors were used as responder cells and were isolated from buffy coats (Finnish Red Cross Blood Service, Helsinki, Finland) by density gradient centrifugation (Ficoll-Paque Premium, GE Healthcare). Isolated PBMNCs were cryopreserved in 10% DMSO (CryoSure DMSO, Wak-Chemie Medical GmbH) and 90% inactivated fetal bovine serum (Gibco, Life Technologies). Responder cells from at least two individual donors were used in each experiment. The responder cells were thawed in +37°C water bath, were let to rest at room temperature for at least 30 min and were subsequently filtered through a 30 μm sterile filter and labeled with 5 μM 5(6)-Carboxyfluorescein succinimidyl ester (CFSE mixed isomers, Molecular Probes, Life Technologies). Co-cultures with MSCs were initiated by first allowing the MSCs to adhere for 3–4 h (max 24 h) before adding the labeled responder cells to the co-cultures together with the activation cocktail composing of 6.5 μg/ml CD3 (anti-human CD3, clone Hit3a, Bio Legend, CA, USA) and 6.5 μg/ml CD28 (anti-human CD28, clone CD28.2, Bio Legend) antibodies. All co-cultures were performed with a T-cell culture medium consisting of RPMI 1640 Medium with L-glutamine and HEPES buffer, 100 U/ml penicillin-streptomycin and 5% inactivated fetal bovine serum (all from Gibco, Life Technologies). Every experiment was performed with replicate samples, at least two different responder cells and also including different responder:MSC cell ratios (1:10, 1:20 and 1:50). Each experiment also contained an IDO inhibition verification with 1.0 mM of the IDO inhibitor 1-Methyl-L-Tryptophan (L-1MT, Sigma). The responder:MSC cell ratio was always 1:10 in the IDO inhibitor tests. The co-cultures were incubated for 4–5 days before flow cytometric analysis of T-cell proliferation (Navios, Beckmann Coulter or BD FACS Aria IIu, Becton Dickinson, NH, USA).

#### Alternative Set-Ups

To elucidate the impact of assay design in the final read-out of the immunosuppression assay, three alternative set-ups were also utilized: the MSCs were either (1) combined directly after thawing with the labeled responder cells and activation cocktail or (2) allowed to adhere to the assay plates for exactly 24 h before adding the labeled responder cells and the activation cocktail (24 h culture rescue), or (3) the responder cells were pre-activated with the CD3/CD28 antibodies for 24 h before adding fresh MSCs from continuous cultures to the assay (*n* = 2).

#### Analysis of Mean Cell Area

A detailed protocol for image acquisition and analysis is described in Oja et al. (25). Briefly, MSCs were plated at density 3000 cells/cm^2^ in 6-well culture plates and were allowed to adhere and proliferate for 48 h before fixation with 4% PFA (Sigma). Fixed MSCs were permeabilized with 0.1% Triton X-100/PBS (Sigma) and the nuclei were stained with 0.125 μg/ml DAPI and the cytoplasm with 1 μg/ml Cell Mask Deep Red Stain (both from Life Technologies). The images were acquired using the Cell Insight high content screening platform (Thermo Scientific, IL, USA). Signals from DAPI and Cell Mask stains were recorded on separate channels, using filters 386 and 630 nm, respectively. Images were acquired from 6 wells resulting in 1998 images per channel on each run and mean cell area in different passages were calculated.

### Statistical Methods

Statistical analyses were performed using the GraphPad Prism (version 7.02) software (GraphPad Software Inc., La Jolla, California). A two-tailed paired *t*-test with a Mann-Whitney test was used for the comparison of two groups. 1way ANOVA with either Bonferroni's multiple comparisons test or Brown-Forsythe test (**Figure 6C**) was utilized when comparing multiple groups. Differences were considered statistically significant when *p* < 0.05.

## Results

### Excellent Viability and Cell Recovery and Restored Basic MSC Characteristics After a Single Freezing Step at Passage 2

**Figure 2:**
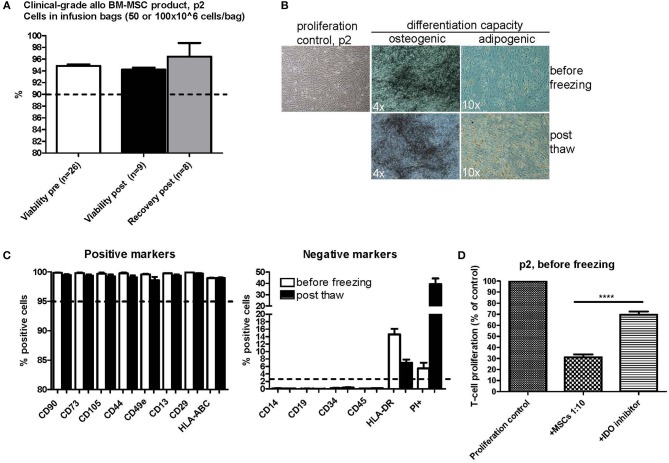
Characterization of clinical-grade allogeneic BM-MSC product in passage 2 before and after one freezing step**. (A)** Viability before freezing (white) and after thawing (black) and cell recovery after thawing (gray). The cells were frozen at passage 2 in freezing bags with either 50 or 100 × 10^6^ cells/bag. Results are presented as mean ± SEM. The viability differences were non-significant (*p*-value 0.1716 with a non-parametric two-tailed *t*-test and 95% confidence interval). **(B)** Osteogenic and adipogenic differentiation capacity of MSCs before and after freezing. Representative phase contrast microscope pictures are presented (*n* = 3). Enlargement is indicated in each picture separately. **(C)** MSC phenotype before (white) and after (black) freezing as studied by flow cytometry. Results are presented as mean ± SEM (*n* = 3). **(D)** T-cell proliferation assay results of clinical-grade MSCs in passage 2 before freezing with IDO inhibition. Results are presented as mean ± SEM (*n* = 16). 1-Methyl-L-Tryptophan (L-1MT) was utilized as an IDO inhibitor. ^****^p < 0.0001.

As part of the stability testing program of our in-house manufactured clinical-grade bone marrow-derived MSC product, which is frozen in p2 (7, 22), we investigated the quality and functional properties of the product before and after the single freezing step at p2. The outlines of the clinical MSC culture protocol is presented in Figure 1A. The cells were frozen in doses of either 50 or 100 × 10^6^ cells in 50 ml freezing bags and were thawed and diluted in 0.9% NaCl according to a validated protocol in use bedside in the clinics, which also includes forcing the cells through a syringe and 200 μm filter (see Materials and Methods section for more details). The viability of 26 individual clinical MSC batches in p2 before freezing is presented in Figure 2A. The mean viability of these batches was 94.8% (min 91.5%, max 96.7%) before dispensing the cells in freezing bags and initiating the freezing. The viability and recovery was studied in 8–9 individual bags (derived from four individual MSC batches) after freezing and thawing. As presented in Figure 2A, the mean viability and cell recovery after thawing was 94.2% (min 92.7%, max 95.4%) and 96.4% (min 88.2%, max 100%), respectively. We can therefore conclude a superior viability and cell recovery after one freezing step in p2 utilizing the freezing and thawing protocols we have developed and validated for large cell amounts (50 or 100 × 10^6^ cells) in 50 ml freezing bags and even after forcing the thawed cells through standard infusion materials composing of a syringe, 200 μm filter and infusion lines.

The osteogenic and adipogenic differentiation potential of cells from p2 was examined before and after freezing. We compared the differentiation potential in at least three individual clinical-grade MSC batches and we always observed a preserved differentiation potential in all of these batches (Figure 2B). The basic MSC phenotype, as studied by flow cytometry, was also compared before and after freezing in the exact same MSC batches (Figure 2C). The generally accepted positive MSC markers CD90, CD73, CD105, CD44, CD49e, CD13, CD29, and HLA-ABC remained unaltered in the flow cytometry analysis directly after thawing (Figure 2C, left panel) and clearly fulfilled the ISCT minimal criteria of ≥95% positive expression (26). The expression of the negative MSC markers CD14, CD19, CD34, and CD45 (Figure 2C, right panel) also fulfilled the ISCT minimal criteria (26) and were consistently ≤2% with the exception of HLA-DR (Figure 2C, right panel). The HLA-DR cell surface expression in pooled platelet lysate cultured BM-MSC has previously been shown to be routinely ≥2% (22). The mean HLA-DR cell surface expression in the 26 individual MSC batches analyzed in this study was 16.2% at p2 before freezing (min 0.9%, max 56.5%) (data not shown). The HLA-DR expression was above the ≥2% expression limit also after freezing and thawing as presented in Figure 2C (right panel), but it is interesting that there was a drop of about 8% in the HLA-DR cell surface expression as compared to the expression levels in the same cell batches before freezing. It is also noteworthy that we observed a substantial increase in PI+ positive cells in the post thaw cell samples (Figure 2C, right panel), indicating that the FACS staining and analysis procedure is harsh on newly thawed cells, since the cell viability (as determined with the NucleoCounter NC-100 method that also measures PI penetration) was concluded to be excellent directly after thawing as presented in Figure 2A.

The *in vitro* immunosuppression capacity of 16 individual clinical MSC batches in p2 before freezing is presented in Figure 2D utilizing a T-cell proliferation assay. The proliferative response was normalized to the proliferation control set as 100% in each assay. Every MSC batch consistently inhibited the proliferation of the activated responder T-cells to a mean of 31.06% of the original proliferation rate (min 13.75%, max 59.93%). Addition of the IDO inhibitor 1-Methyl-L-Tryptophan (L-1MT) significantly inhibited the immunosuppression capacity of the MSCs by approx. Fifty percent resulting in a mean T-cell proliferation rate of 69.52% of the proliferation control (min 37.58%, max 92.50%) (*P*-value < 0.0001 with a two-tailed paired *t*-test) (Figure 2D). The immunosuppression capacity of frozen and thawed MSCs is presented in **Figures 4**, **5**.

### Introducing an Additional Interim Freezing Step Does Not Affect Basic MSC Manufacturing Parameters

**Figure 3:**
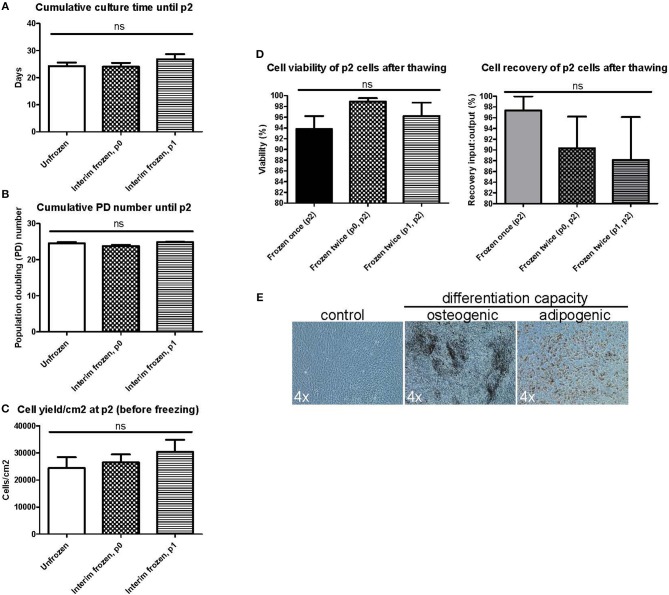
Manufacturing parameters and characterization of BM-MSCs in passage 2 after two freezing steps. MSC products in passage 2 (p2) are compared after no preceding freezing steps (unfrozen), one freezing step at passage 0 (p0) or one freezing step at passage 1 (p1). **(A)** Cumulative culture time at p2. **(B)** Cumulative population doubling (PD) time. **(C)** Cell yield per cm^2^ at p2 before freezing. **(D)** Cell viability (left) and cell recovery (right) at p2 after freezing and thawing. **(E)** Osteogenic and adipogenic differentiation capacity of MSCs frozen twice in either p0 or p1 and p2. Representative phase contrast microscope pictures presented from *n* = 3. Enlargement indicated for each picture separately. Results in **(A–D)** are presented as mean ± SEM (*n* = 4).

We next wanted to explore the possible impact of introducing additional interim freezing steps at either p0 or p1 in addition to the final freezing step at p2 (Figure 1B). No differences were seen in the cumulative culture time (Figure 3A), the cumulative population doubling (PD) number (Figure 3B) or the cell yield/cm^2^ (Figure 3C) until reaching p2 when comparing unfrozen MSCs initiated from fresh bone marrow to MSCs frozen at p0 or p1 and subsequently subcultured until p2. Unfrozen and interim frozen cells from the same MSC batches were compared in these experiments and the experiments were repeated with four individual MSC batches. No statistically significant differences could be observed in either the p2 cell viability or the recovery after thawing utilizing only one freezing step (control) or two freezing steps either at p0 + p2 or p1 + p2 (Figure 3D). The osteogenic and adipogenic differentiation capacity was also clearly not affected by an additional freezing step and the differentiation results of p2 post thaw cells after two freezing steps (Figure 3E) were completely comparable to the differentiation potential after one freezing step and, importantly, directly comparable to the differentiation capacity of unfrozen MSCs (Figure 2B).

### Impaired *in vitro* Immunosuppression Activity of Thawed MSCs

**Figure 4:**
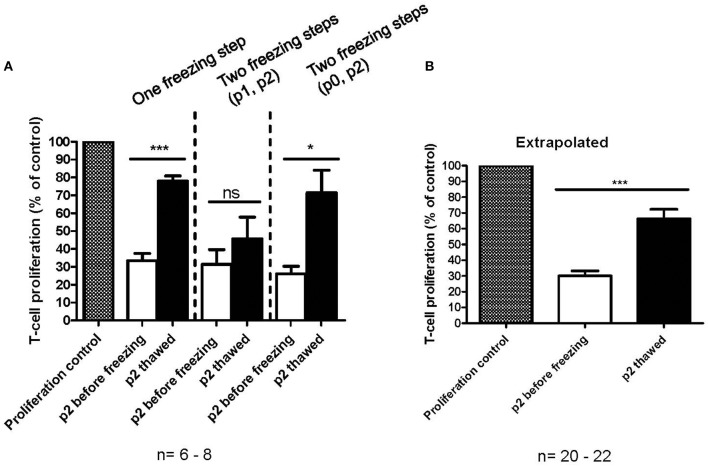
*In vitro* immunosuppression capacity of cryopreserved MSCs as measured by a T-cell proliferation assay. **(A)** Immunosuppression capacity of MSCs before (white) and after freezing and thawing (black) with one freezing step at passage 2 (p2) or two freezing steps at either p0 or p1 and p2. The results are presented as mean ± SEM (*n* = 6-8). **(B)** Comparison of fresh (white) and frozen and thawed (black) MSCs in p2. The data in extrapolated from all the data presented in **(A)**, regardless of the number of freezing steps. The results are presented as mean ± SEM (*n* = 20–22). ^*^p < 0.05, ^***^p < 0.001.

The immunosuppression capacity of MSCs before freezing and after one freezing step at p2 or two freezing steps (p0 + p2 or p1 + p2) was studied primarily using a T-cell proliferation assay (Figures 4A,B). The results indicate that the immunosuppression potential of frozen and thawed MSCs is reduced by approx. Fifty percent (*p* < 0.0001, *n* = 20) as compared to the fresh counterpart, at least in this particular *in vitro* assay design for immunosuppression (Figure 4B). It is, however, evident that additional interim freezing steps at p0 or p1 do not affect the immunosuppression potential of the final product at p2 (Figure 4A) and, interestingly, the difference in immunosuppression before and after freezing is not as evident and even not statistically significant in cells after two freezing steps. It should be pointed out that the MSCs were introduced to the FCS containing immunosuppression assay medium when initiating the assay, which is also the situation for the thawed MSCs. It should also be pointed out that the immunosuppression potential of frozen and thawed MSCs was not completely abolished, but was merely reduced by approx. Fifty percent to approximately 66% of the proliferation control (Figure 4B). Interestingly, the immunosuppression potential of frozen and thawed MSCs is comparable to the immunosuppression assays with fresh MSC where the IDO inhibitor 1-Methyl-L-Tryptophan (L-1MT) was added as presented in Figure 2D.

### Immunosuppression *in vitro* Assay Design and Impact On Readout

**Figure 5:**
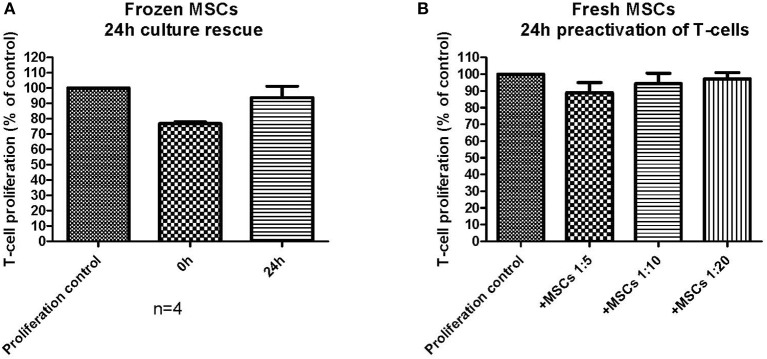
The impact of assay design and the performance of an *in vitro* immunosuppression assay. **(A)** Frozen and thawed MSCs were either combined directly (0 h) with activated T-cells or pre-plated (culture rescued) for 24 h before combined with the activated T-cells (*n* = 4). **(B)** Fresh MSCs were combined with T-cells, which had been pre-activated for 24 h before combining with the MSCs. The results are presented as mean ± SEM.

The chosen primary immunosuppression assay used in this study was a T-cell proliferation assay, where the MSCs were always first plated alone and allowed to adhere (usually 3–4 h and up to 24 h with fresh MSCs), after which the labeled responder MNCs were added to the assay together with the T-cell activator molecules CD3 and CD28. Fresh MSCs consistently inhibited the T-cell proliferation with all studied MSC batches in this assay format with a variable initial MSC adhering time from 3–24 h (Figure 4). Since the frozen and thawed MSCs were not allowed to adhere for more than 9 h, we next wanted to confirm that the reduced immunosuppression of the frozen and thawed MSCs (Figure 4) were not due to a shorter adhering time. In Figure 5A, frozen and thawed MSCs were either combined with the responder cells directly (0 h) after thawing or after a 24 h adherence time. This 24 h adhering period can alternatively be named a 24 h culture rescue period as used by others (11). As can be seen in Figure 5A, a 24 h rescue period did not improve the *in vitro* immunosuppression response and, on the contrary, almost completely diminished the *in vitro* immunosuppression response of thawed MSCs. The immunosuppression of thawed MSCs immediately combined with the responder cells (0 h) was completely comparable to the frozen and thawed MSCs after 3–9 h of preplating as presented in Figure 4. In our hands and with the chosen assay format, we could not see a benefit of a 24 h culture rescue period for the frozen and thawed MSCs.

We further wanted to explore how small changes in the immunosuppression assay design could possibly impact the final result. In the basic immunosuppression assay format, we activate the responder MNCs simultaneously when added to the MSCs. We therefore explored the impact of utilizing pre-activated responder MNCs with fresh MSCs (Figure 5B). The result was strikingly different: the fresh MSCs, which always perform well in the *in vitro* immunosuppression assay, produced only a very modest immunosuppression, even in higher cell:responder ratios, and the immunosuppression was even worse than for the frozen and thawed MSCs presented in Figure 4. This experiment demonstrates well how easily an immunosuppression readout can be modified by small, but critical, changes in the assay design.

### The Impact of Freezing on Replicative Senescence

**Figure 6:**
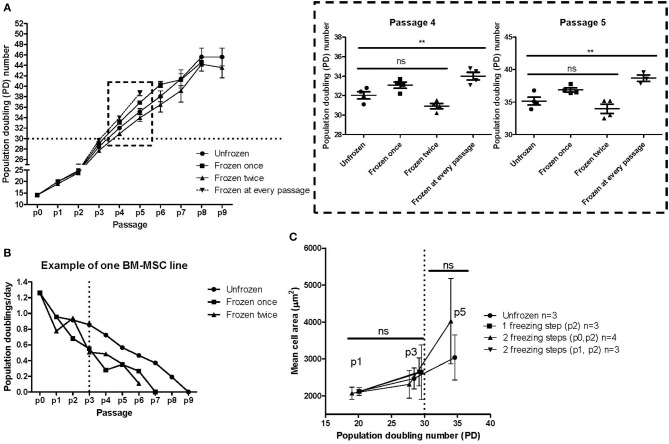
MSC freezing and impact on replicative senescence. **(A)** Cumulative population doubling (PD) numbers at each passage of the same MSC line without any freezing steps, one freezing step, two freezing steps or freezing at every passage until reaching replicative senescence. The results are presented as mean ± SEM of four individual MSC lines. The box with the dashed boarder lines containing the results for passage 4 and passage 5 is enlarged on the right. **(B)** Example of the growth kinetics of one of the four individual MSC lines presented in Figure 6A. **(C)** Mean cell area compared to population doubling (PD) numbers of either unfrozen MSCs or after either one or two freezing steps. ^**^p < 0.01.

Four individual MSC lines derived from individual bone marrow donors were cultured from fresh bone marrow and with continuous passaging until reaching complete replicative senescence (Figure 6A). During these culturing experiments, aliquots were taken during passaging and were cryopreserved (see experiment layout in Figure 1C). Frozen interim samples were subsequently thawed and cultured until senescence. We finally compared the culture kinetics of the same individual MSC line without any freezing steps, one freezing step, two freezing steps or freezing at every passage until reaching p5 (Figure 6A). We chose to analyze the data by comparing the population doubling (PD) numbers at each passage vs. the passage numbers. A strikingly similar culture kinetics with no statistically significant differences at any passage number was observed between the unfrozen cultures vs. cells that had been frozen once (at p1) or twice (at p0 and p2). We can, however, conclude that there is more variability in the culture kinetics after higher passaging numbers >p3. Interestingly, some significant differences were revealed in the cultures where the MSCs were frozen at every passage from p0 until p5, where the cells that were frequently frozen and thawed clearly had a higher PD number compared to cells at the same passage number which had either never been frozen or had been frozen once or twice during culturing (Figures 1C, 6A, blow up). This was, however, evident only at the higher passage numbers 4 and 5 and was not still evident at passage 2 or 3. This might indicate that freezing and thawing affects the senescence mechanisms, but only after an exhaustive number of freezing steps. Furthermore, the possible impact of freezing steps might be very individual for a particular MSC line, since one of the four analyzed MSC lines clearly demonstrated a different culture kinetics after one or two interim freezing steps as compared to the unfrozen culture (Figure 6B). In this particular case, it looked like this individual MSC line reached senescence (defined as when the number of PD's/day reached zero) two passages earlier (Figure 6B). It is also evident that the PD capacity per day decreases as early as from p2-3, but markedly after p3 (Figure 6B). We finally looked at the mean cell area correlated to both passage and PD numbers as an indicator of early senescence (Figure 6C). The cell area is clearly unaffected after one to two freezing steps as compared to their unfrozen counterparts during early passage up to p3 and PD numbers up to 30 (Figure 6C). At later passage numbers the variability in cell size increases, but the cell size differences between cells that have been frozen twice and their unfrozen counterpart is unsignificant (Figure 6C), which indicates that a moderate number of freezing steps does not significantly affect early onset of senescence of MSCs.

## Discussion

The conflicting results on the potential functional consequences of MSC cryopreservation led us to thoroughly investigate the functionality and quality attributes of our clinical-grade MSC product, mainly by utilizing a set of standard quality control and batch release assays used in clinical MSC manufacturing by us and others. Our in-house GMP manufacturing protocol for an allogeneic BM-derived platelet lysate-expanded MSC product contains only one freezing step at p2 (Figure 1A). The cryopreserved MSC products in p2 are subsequently provided frozen to the clinics when needed for the treatment of refractory acute GvHD and are thawed bed-side immediately before i.v. administration according to a published standard operating procedure (7, 22). In favor of MSC cryopreservation, we report in this study (and as part of our MSC product stability program) a 94.2% viability and 96.4% recovery for the frozen and thawed MSC product in p2, which to our knowledge is superior to other published reports (11, 12, 27, 28). We can also conclude that the basic MSC cell phenotype for platelet lysate-expanded MSCs and the osteogenic and adipogenic differentiation potentials are unaltered after freezing and thawing. Furthermore, introducing additional interim freezing steps at p0 and p1 does not alter the viability, recovery, cumulative PD number, cell yield, cell phenotype or differentiation potential of the frozen and thawed MSC product in p2, which is in line with an earlier study performed with research-grade MSCs (29). When comparing PD numbers between different studies it should be noted that our PD numbers, unlike most other studies, are calculated starting from the original CFU-F numbers which thus also includes the first cell divisions of the original MSCs in the primary culture. Our cumulative PD numbers demonstrate that on average 14 population doublings actually take place during the first cell culture stage in p0, which is usually disregarded in most other studies.

An additional conclusion made from this study, and in favor of MSC cryopreservation, is that we cannot see a clear correlation between a modest number of cryopreservation steps and earlier induction of senescence. Our data, however, give reason to believe that a) the induction of senescence is highly heterogenous between individual MSC batches (derived from different donors) and that b) exhaustive numbers of freezing steps (≥4) might induce earlier onset of senescence. We made a choice to mainly utilize a set of standard quality control and batch release assays for clinical MSC products. We must, therefore, point out that we did not analyze the impact of MSC cryopreservation on a molecular level, where the effect of a stressful cell handling procedure (which cryopreservation and thawing most certainly is) may be more evident (12, 16). It is nevertheless unclear whether transient changes on a molecular level will ever translate to a functional level that really ultimately affect cell functionality or cell performance. We cannot, however, emphasize enough the importance of utilizing process developed and fully validated protocols for MSC freezing and thawing combined with fixed, unambiguous standard operating procedures and documentation of every thawing procedure also at the clinical site. This may in the end be the most relevant parameter for the quality of frozen and thawed MSCs.

The crucial question however still remains: does cryopreservation negatively affect the immunomodulatory potential of MSCs as demonstrated in some studies (11, 12)? We tried to address this question by an *in vitro* immunosuppression assay currently in use as a potency assay by us in our in-house clinical-grade MSC batch release. Our immunosuppression assay utilizes PBMNCs derived from allogeneic blood donors as responder cells. A single well in this assay only contains PBMNCs from a single donor, as opposed to a mixed lymphocyte reaction (MLR). The T-cells in the PBMNC fraction are activated with CD3 and CD28 antibodies and the MSCs are not irradiated, as usually is the case in an MLR setup. Our assay format consistently displays a potent immunosuppressive *in vitro* behavior of fresh MSCs with a 70% inhibition of T-cell proliferation at a 1:10 ratio (Figure 2D). It is also evident that this particular assay measures immunosuppression linked to the IDO pathway, since the addition of the IDO inhibitor L-1MT reduces the MSC-induced T-cell proliferation inhibition by over 50% (Figure 2D). It is, however, clear that frozen and thawed MSCs do not perform as well as their unfrozen counterparts in this assay (Figure 4). When extrapolating all the immunosuppression data we have from frozen and thawed MSCs, regardless of the number of freezing steps before p2, we can conclude that the immunosuppressive potential is reduced by ~50% as compared to the fresh counterpart (Figure 4B). Strikingly, the result is almost identical to the IDO inhibitor L-1MT result (Figure 2D). Since we made an observation that the assay performance was somewhat inconsistent in the experiments comparing fresh and frozen cells after one or two freezing steps (Figure 4A), where we even saw some frozen MSCs performing almost as well as fresh cells, we were challenged to critically evaluate the design of our assay and in particular the assay compatibility with frozen and thawed MSCs. As we exemplify in Figure 5, the assay readout is highly dependent on what might be considered “insignificant experimental details” such as plating times of the MSCs and the activation protocol of the T-cells. It is striking that changing the activation protocol to a 24 h pre-activation of the T-cells before MSC addition dramatically changes the outcome and even fresh MSCs did not perform in our assay anymore (Figure 5B). It is also interesting that we could not restore the immunosuppressive potential with a 24 h rescue period/plating period after thawing in Figure 5A as demonstrated by François et al. (11).

Our results ultimately forces one to ask the question: what does our *in vitro* immunosuppression assay measure? Our assay format is standardized, but it differs significantly from an MLR assay, where PBMNCs from two unrelated donors are mixed to induce the allostimulation of T-cells. This assay can to some degree measure the proliferation of activated CD3+ T-cells and an IDO-pathway related immunosuppression, but it is clear it does not directly address the effect of MSCs on other cellular components of PBMNCs such as B cells, regulatory T-cells, dendritic cells and monocytes and macrophages (30, 31). The assay does not either measure other alternative MSC immunosuppressive pathways such as the adenosinergic pathway by ectonucleotidases CD73 and CD39 (32). The adenosinergic immunosuppressive pathway has interestingly been shown to be an important immunoregulatory mechanism of MSCs in situations where extracellular ATP is available in excess, such as in tissue injury (32). Furthermore, the exciting MSC immunomodulation paradigm shift recently presented by Galleu et al. (9) demonstrates that MSCs undergo extensive caspase activation and apoptosis after infusion in the presence of cytotoxic cells. After infusion, recipient phagocytes engulf apoptotic MSCs and subsequently produce IDO after polarization to M2 macrophages, which is ultimately necessary for effecting immunosuppression. de Witte et al. furthermore demonstrated that both CD8+ T-cell and CD56+ natural killer (NK) populations are responsible for initiating MSC apoptosis (10). This mechanism has also been supported by clinical data, whereby only those GvHD patients with high cytotoxic activity against MSCs responded to MSC infusion, and those GvHD patients with low activity did not respond to MSC therapy (9). This new apoptosis-based MSC immunomodulatory mechanism challenges the design of *in vitro* immunosuppression assays even further, since the assay should also contain functional cell types to induce MSC apoptosis and, importantly, functional monocytes that can polarize to M2 macrophages. It has also been recently presented that an optimal *in vitro* immunosuppression assay should not allow MSCs to adhere to plastic to avoid baseline activation of monocytes adhering to the plastic and for instance polypropylene tubes should be utilized instead in order to more closely resemble the *in vivo* setting (10). Another evident weakness in the performance of an *in vitro* immunosuppression assay might be the utilization of fetal calf serum (FCS) in the T-cell expansion media. In our case, platelet lysate-expanded MSCs encounter FCS for the first time in the immunosuppression assay. We have thus far been unsuccessful in finding a xenofree substitute for FCS with equal performance in this assay format. Utilizing FCS in the immunosuppression assay does not cause obvious evident problems for fresh platelet lysate-expanded MSCs. The xenogeneic stress might be tolerable for fresh MSCs, but when a cell is simultaneously recovering from a thawing procedure, the xenogeneic stress caused by FCS might impact the performance of the MSCs in the assay. Luetzkendorf et al. demonstrated preserved immunomodulatory functions of their platelet lysate expanded cryopreserved MSCs in an FCS-based assay, but by utilizing mitogen (phytohemagglutinin; PHA)-stimulated PBMNCs and a readout based on bromodeoxyuridine (BrdU) incorporation (27). Furthermore, Kuci et al. convincingly demonstrated a clear *in vitro* immunosuppressive result for their frozen and thawed clinical-grade MSC product MSC-FFM, but by a two-way MLR assay (20). It is, however, also evident that the Frankfurt platelet lysate-expanded MSC-FFM product is manufactured in a very different manner from ours utilizing pooled BMNCs from multiple donors to produce the MSCs (20, 21), which might significantly impact the immunomodulatory performance, also *in vitro*.

As discussed above, the weakness of our study may lie in the design of the immunosuppression assay, or more precisely, in the utilization of only one assay format (31). Furthermore, we did not measure cytokines in the assay media (31). It is, however, an inevitable fact that *in vitro* functionality data can only answer questions concerning efficacy to a limited degree. In the light of our immunosuppression results, it is evident that the cryopreserved and thawed MSCs perform differently from the fresh counterpart, but that does not necessarily translate to reduced immunomodulatory efficacy *in vivo*. The ultimate answers therefore lie within the clinical data and unless the MSC product is rigorously evaluated and demonstrated to correlate with clinical outcomes, the *in vitro* immunosuppression assay can only be used as a research tool. Our clinical-grade MSC product has been in clinical use since 2013 and the results of the first aGvHD patient cohort consisting of 26 adult and pediatric patients, most with grade III-IV GvHD, have been published earlier (7). We can conclude an overall response rate of 62% at day 28, but with a disappointing overall survival of only 22% after a median follow-up time of 767 days (range 74-1270 days) (7). Our results also presented a markedly different overall long-term survival rate between adult and pediatric patients and between complete responders and non-responders. Thus far, we have been unable to find molecular markers that can predict the outcome or find a mechanistic explanation to the variable degree of response (8). Several other MSC GvHD patient cohorts have been published lately utilizing a similar MSC manufacturing strategy including cryopreservation and supplementing the culture media with pooled platelet lysate (21, 33–37). When comparing our published clinical cohort with the six other published MSC GVHD cohorts utilizing platelet lysate-expanded and cryopreserved MSCs, it can be concluded that although the overall response rate at day 28 is to some extent comparable (ranging from 47–83%), there is a large difference in the reported overall survival rates ranging from 16.6–67% (follow-up times from 6 months to >2 years). The very variable follow-up times in the published patient cohorts so far and the mixed degree of adult vs. pediatric patients complicates the study comparisons. The obvious limitation in all these cohorts is the seemingly small number of patients included and the lack of control groups and randomization. The very recent publication by the Frankfurt am Main group presents the largest patient cohort of 69 patients, but mostly consisting of pediatric patients (74%) (21). The Frankfurt cohort presents superior overall response rates of 83% at day 28 and a very promising overall survival rate of 67%, but it needs to be pointed out that the mean follow-up time was only 8.1 months and the clinical monitoring needs to be continued. Nevertheless, the Frankfurt overall response rates and preliminary survival data is superior to the other MSC GvHD cohorts with frozen, platelet lysate-expanded MSCs. The Frankfurt MSC-FFM manufacturing strategy differs significantly from the other studies by utilizing a pooled BM-MNC bank derived from eight allogeneic donors as a source for bulk production of the clinical-grade MSC products (20), which naturally might result in a more immunosuppressive product, but the MSC-FFM product is cryopreserved and the manufacturing strategy includes at least two freezing steps. It is therefore tempting to speculate, in light with the new apoptosis-based theory (9), that cryopreserved MSCs might in fact cause a stronger immunosuppression since it has been demonstrated that freeze-thawed MSCs are more prone to activate the instant blood mediated inflammatory reaction (IBMIR) and display increased sensitivity to complement lysis, which leads to increased MSC apoptosis (12). This is in-line with another study demonstrating that cryopreserved MSCs are significantly more susceptible to contact-dependent apoptosis when co-cultured with activated T-cells (13). The long-term survival data of patient cohorts utilizing cryopreserved MSC products will provide more insights into what parameters might cause differences in the outcome, but the available published clinical data suggest that the functional consequences of cryopreservation might be inconsequential as compared to other MSC manufacturing and administration details.

## Conclusions

Our results support the utilization of frozen MSC products and MSC banking strategies and suggest that 1-2 freezing steps for MSCs in early passage is feasible and preserves most of the *in vitro* functional properties. Interim freezing steps are not reflected in standard manufacturing parameters. The *in vitro* immunosuppressive potential of frozen and thawed MSCs can be interpreted as different from the fresh counterpart, with a reduced, but definitely not abolished *in vitro* performance specific for the IDO pathway, but *in vitro* immunosuppression assay results ought to be interpreted with caution to avoid false conclusions. Our study emphasizes the need of always performing detailed studies on also the cryopreserved MSC counterpart and to thoroughly validate both the freezing and thawing procedures and to monitor the thawing procedures with unambiguous standard operating procedures. All MSC products are not equal, and frozen-thawed MSCs might differ to some extent to their fresh counterpart. This, however, does not mean that cryopreserved MSCs are not efficacious, they just might work differently from fresh MSCs. In light of available clinical data, it is tempting to speculate that the ultimate parameter affecting efficacy might not actually be cryopreservation, but essential differences in MSC manufacturing strategies.

## Data Availability

All datasets generated for this study are included in the manuscript and/or the supplementary files.

## Author Contributions

SO planned and performed the majority of the experiments and assisted in the results analysis and manuscript writing. TK and AL performed some of the experiments, assisted in the results analysis and contributed to the interpretation of the results. MA performed some of the experiments and assisted in results analysis. MK provided study materials, supported the supervision of the study and contributed to the interpretation of the results. JN conceived the original idea, planned the experiments, supervised the study, analyzed the results, and wrote the manuscript. All authors have commented and accepted the manuscript.

### Conflict of Interest Statement

The authors declare that the research was conducted in the absence of any commercial or financial relationships that could be construed as a potential conflict of interest.
